# Gut Microbiota Variation in Aging Dogs with Osteoarthritis

**DOI:** 10.3390/ani15111619

**Published:** 2025-05-30

**Authors:** Fatemeh Balouei, Christina de Rivera, Andrea Paradis, Bruno Stefanon, Stephanie Kelly, Noelle McCarthy, Paolo Mongillo

**Affiliations:** 1 Agrifood, Environmental and Animal Science, University of Udine, Via delle Scienze, 206, 33100 Udine, Italy; balouei.fatemeh@spes.uniud.it; 2 Transpharmation Canada Ltd., Fergus, ON N1M 2W8, Canada; christina.derivera@transpharmation.com (C.d.R.); andrea.paradis@transpharmation.com (A.P.); stephanie.kelly@transpharmation.com (S.K.); noelle.mccarthy@transpharmation.com (N.M.); 3 Department of Comparative Biomedicine and Food Science, University of Padova, Viale dell’Universita’ 16, 35020 Legnaro, Italy; paolo.mongillo@unipd.it

**Keywords:** gut microbiota, dogs, aging, osteoarthritis, 16S rRNA gene amplicon sequencing

## Abstract

Gut microbiota composition plays a crucial role in host health and may be influenced by age and disease conditions. This study investigates the gut microbiota diversity of 175 dogs across three age groups (Junior (20–46 months, 43 dogs), Adult (47–92 months, 58 dogs), and Senior (93–168 months, 74 dogs), and examined the impact of osteoarthritis on microbial composition. Alpha diversity analysis using the Shannon and Chao1 indices were significant (*p* < 0.05) in Senior dogs Beta diversity analysis based on Bray–Curtis dissimilarity indices demonstrated substantial overlap in gut microbiota composition across age groups, with no significant clustering observed (*p* > 0.05). A second analysis compared the microbiota of 69 healthy dogs and 81 dogs affected by osteoarthritis (OA) in the three classes of age. No significant differences were shown for alpha diversity and beta diversity between healthy and OA dogs. This indicates that aging and osteoarthritis do not induce significant shifts in microbial beta diversity, although high inter-individual variability was noted. Linear Discriminant Analysis (LDA) Effect Size (LEfSe) analysis identified distinct bacterial taxa associated with different age groups. Linear Discriminant Analysis (LDA) Effect Size (LEfSe) analysis identified distinct bacterial taxa associated with different age groups. Junior dogs exhibited enrichment in *Blautia*, Erysipelotrichaceae, and *Clostridium*, while Adult dogs were characterized by higher abundances of *Prevotella*, *Streptococcus*, and Ruminococcaceae. Senior dogs had increased representation of *Prevotella* and *Ruminococcus*. In OA dogs, *Peptococcus*, *Peptostreptococcus*, Clostridiaceae, and *Coprobacillus* were significantly enriched in comparison to healthy dogs, suggesting potential microbiota shifts associated with osteoarthritis. Overall, these findings indicate that gut microbiota diversity varies across different life stages, specific bacterial taxa were differentially enriched in relation to age and OA. This study enhances our understanding of gut microbiota dynamics in dogs and provides insights into potential age- and disease-related microbial signatures.

## Simple Summary

This study examined the gut microbiota of 175 Beagle dogs living in a colony across three age groups Junior (43 dogs), Adult (58 dogs), and Senior (74 dogs) and assessed the impact of osteoarthritis on microbial composition. Shannon and Chao1 alpha diversities were significantly lower in Senior dogs, but not beta diversity. No differences were shown between a subgroup of 69 healthy and 81 osteoarthritic dogs. Specific bacterial taxa varied by age. Junior dogs had more *Blautia*, Erysipelotrichaceae, and *Clostridium*; Adults showed higher levels of *Prevotella*, *Streptococcus*, and Ruminococcaceae; and Seniors had more *Prevotella* and *Ruminococcus*. In dogs with osteoarthritis, *Peptococcus*, *Peptostreptococcus*, Clostridiaceae, and *Coprobacillus* were enriched in comparison to healthy dogs. These findings suggest that gut microbiota can vary across life stages and that certain bacteria are more common at different life stages or in dogs with OA. This enhances understanding of gut microbiota patterns in dogs and highlights possible microbial markers of age and disease.

## 1. Introduction

A growing number of studies are exploring the various factors that influence the intestinal microbiota [1,2,3,4]. These factors can be broadly categorized into external and internal influences, such as diet and pharmaceutical treatments for external factors, and age and genetics for internal factors. Moreover, certain pathological conditions, including inflammation and type 2 diabetes, can lead to alterations in the gut microbiota, resulting in an imbalance known as dysbiosis. Among these factors, age is considered one of the most significant variables when examining the development and changes in the gut microbiota over time, which are believed to be linked to age-related diseases [5,6].

The relationship between aging, gut health, and gut microbiota is currently being investigated in mammals, with well-documented findings in humans. Badal et al. [7] reviewed existing knowledge on the human gut microbiota, examining its composition, function, and metabolic products in the context of aging and lifespan. Age-related changes in the human microbiota have been observed and appear to be linked to the host’s overall health. In older individuals, microbial diversity tends to decrease, with an increased presence of Bacteroidetes and a reduced abundance of *Bifidobacterium*, Firmicutes, and Clostridium cluster IV when compared to younger individuals [5,8]. In contrast, *Clostridium perfringens*, Lactobacilli, Enterococci, and Enterobacteriaceae show an increase with aging [9].

Companion animals, especially dogs, serve as valuable models for studying microbiomes in relation to aging. They have relatively long lifespans and develop age-related health issues under living conditions similar to those of humans. Unlike in humans, *Bifidobacterium* may not have a significant role in canine gut health. Findings by Masuoka et al. [9] indicated that Bifidobacteria were present in only half of the youngest dogs examined and were absent in Adult dogs. However, methodological differences should be taken into account when interpreting these results. Garrigues et al. [10] recently examined the development of gut microbiota in the early stages of canine life, highlighting changes in bacterial communities from day 2 after birth up to 52 weeks. By the second day, microbial richness begins to increase, and between days 2 and 21, the initial dominance of Bacillota shifts to a codominance of Bacteroidota, and Fusobacteriota [11]. During the first few weeks of life, puppies exhibit an immature microbiota, characterized by a higher dysbiosis index (DI), an increased presence of *Clostridium difficile*, and a lower abundance of *Clostridium hiranonis* compared to Adult dogs [12]. Around four to six months of age, the microbiota transitions to a composition similar to that of Adult dogs and remains relatively stable throughout adulthood [10].

The microbiome has emerged as a compelling factor that may integrate multiple influences, including aging, especially considering recent findings highlighting the presence of a low-grade chronic inflammatory state in osteoarthritis (OA) [13]. Furthermore, it is widely recognized that OA development is influenced by the interaction of these three key factors: genetic predisposition, aging, and environmental influences [14]. In dogs, osteoarthritis often begins early in life due to developmental joint conditions such as hip dysplasia. Additionally, joint injuries are another significant cause of osteoarthritis in this species [15,16]. With approximately 9 million pet dogs in the UK [16] and 63.4 million households in the US owning a dog [17], the global burden of OA in dogs is substantial, posing a serious threat to canine welfare. In North America, OA is estimated to affect 20% of dogs over the age of one, based on data from 200 veterinarians [18]. Prevalence estimates for OA in the UK dog population vary significantly, likely due to differences in reporting methods. Estimates range from 2.5% to 6.6% for dogs of any age and breed attending primary-care practices [19,20], with figures rising to 20% for dogs over one year old [21]. Beyond its impact on canine welfare, OA presents significant challenges for veterinarians, owners, and breeders worldwide. Due to its chronic, progressive, and potentially debilitating nature, OA can negatively affect the quality of life of both dogs and their owners [22,23,24]. This condition can also affect owners’ well-being, as managing OA in dogs often involves substantial financial costs [25]. Cachon et al. [26] published consensus guidelines for treating canine osteoarthritis (OA) using the COAST staging tool, excluding radiography. These recommendations, based on evidence and clinical experience, aim to provide veterinarians with a practical reference for stage-specific OA management. The goal is to support informed treatment decisions and personalized care.

The connection between intestinal microbiota, gut health, and the aging process in older dogs remains largely unexplored. Further research is essential to unravel this complex relationship and to identify potential strategies for enhancing the aging experience in dogs through gut health interventions. This study sets out to examine variations in intestinal microbiota composition in 175 Beagle dogs living in a colony, grouped in Junior (43 dogs), Adult (58 dogs) and Senior (74 dogs). Furthermore, a second aim was to investigate microbiota in relation to osteoarthrosis (OA). Since all Junior dogs were healthy, the comparison was carried out between 26 healthy and 23 OA Adult dogs and 9 and 52 Senior dogs. Gut microbial markers were analyzed to gain deeper insights into how aging influences the gut environment, ultimately aiming to contribute to the development of targeted approaches for promoting healthier aging in canine populations.

## 2. Materials and Methods

### 2.1. Animal Ethics Statement

The study facility complied with local regulations for the care and use of laboratory animals. Procedures were designed to prevent or minimize discomfort, distress, and pain to the animals in accordance with the Animals for Research Act of Ontario (ON, CA) and the guidelines of the Canadian Council on Animal Care (CCAC). The CCAC Guide for the Care and Use of Experimental Animals and related policies were regarded as guidelines to follow. The protocol was reviewed and approved by the study facility’s Institutional Animal Care and Use Committee (IACUC) before the start of the trial, as per IACUC standard operating procedures.

### 2.2. Animals and Diets

All Beagle dogs were from the Transpharmation Canada Ltd. (Fergus, ON, Canada) colony. All dogs from the colony had fecal samples collected unless their study treatment might have influenced the microbiome or if collection interfered with sponsored study objectives. Dogs were fed using a standard commercial kibble (Purina^®^ ProPlan^®^ All Ages Sport Active 27/17 Chicken & Rice Formula) for the duration of the study. Dogs were individually fed once daily to maintain body condition according to standard operating procedures. Feed was offered in stainless-steel bowls at the end of each day. Water was provided ad libitum (animals could drink water at any time, without restriction) via automatic sippers or stainless-steel bowls. The water source was an on-site well. For the study, 175 Beagle dogs, 98 females and 77 males, were involved in the research and the inclusion criteria were without pharmacological treatments, specifically no NSAIDs, antibiotics, corticosteroids or deworming drugs during the 30 days before the sampling. The medical history and records were available for each dog, as well as body condition score (BCS) and age. None of the dogs presented watery, soft stools or clinical signs of gastrointestinal disease at the time of sampling. Animals were housed in kennels alone or in two individuals, depending on the size of the box. The area for each dog was at least 15 m2. The animals had free access to water and were allowed access to an outdoor area for 2 to 4 h for socialization with other dogs. The diet was a Purina kibble, fed to the amount indicated by the manufacturing, to cover the nutrient requirements of the National Research Council [27].

Table 1 provides an overview of a population of 175 dogs, categorized by age group, sex, and health conditions. The data distinguishes between spayed females (Fs) and castrated males (Mc) and categorizes health conditions into three groups: Healthy, osteoarthritis, and other diseases. The mean and standard deviation of live weight of the dogs was 10.2 ± 1.7, 11.5 ± 1.8 and 10.7 ± 1.3 for Junior, Adult and Senior, respectively.

Table 2 presents data on a subset of 136 dogs categorized by age group and health condition (healthy or osteoarthritic). The “other” disease category from Table 1 was not included here, focusing solely on healthy vs. osteoarthritic dogs. The new groups were Junior healthy (Junior_HE; n = 26), Adult healthy (Adult_HE, n = 26), Senior healthy (Senior_HE, n = 9), Adult osteoarthritic (Adult_OA, n = 23) and Senior osteoarthritic (Senior_OA, n = 52).

### 2.3. Data Recording and Samples Collections

Each dog was placed in a metabolic cage or individual pen to facilitate fecal collections. Animals were isolated for collections in the evening after being fed. Each dog was put into individual housing for up to 1 h following feeding. After 1 h, the metabolic cage was inspected for a naturally voided sample. If a sample had been produced, it was collected, and the animal was returned to its home pen. The metabolic cage was then cleaned, and another animal was placed into the same metabolic space for overnight collections. If a sample had not been produced, the animal remained in individual housing overnight.

For up to 4 days and 3 nights, the procedure was repeated, and each dog was placed in a metabolic cage or isolated pen until a fecal sample was obtained. Overnight samples were collected the following morning and documented accordingly. Animals returned to their home pens each morning. If fecal samples were not produced, dogs were walked post-feeding to encourage defecation or monitored within their home pens.

Using a gloved hand, the fecal sample was picked up and placed on clean weigh paper. The sample collected had the outer edges removed using a clean plastic knife to minimize bacterial growth. The center of the sample was divided into three aliquots of 250 mg each. Each aliquot was weighed using clean weigh paper that had been tared on the scale and was transferred using a clean plastic knife per sample. Each tube was inverted to ensure the sample was well mixed.

Aliquots were placed into three separate tubes: two ZYMO tubes (brown top) and one eNAT tube (blue top). Tubes were labeled with the animal ID, the date, and the time of fecal collection.

These tubes were stored in a refrigerator at 2–8 °C until shipping. Aliquot 1 was shipped to the bioanalytical laboratory at the address below. Aliquots 2 and 3 remained in Transpharmation Canada Ltd (Fergus, ON, CA). storage until sample analysis.

### 2.4. Microbiota Analysis

DNA extraction was performed within 6 weeks from the collection date. Total DNA extraction for microbiome analysis was performed within 6 weeks from the collection date on 150 mg of feces using the Quick-DNA™ Fecal/Soil Microbe Miniprep Kit (Zymo Research, Irvine, CA, USA), according to the manufacturer’s instructions. Quantification and quality check of the DNA were carried out using a QubitTM 3 Fluorometer (Thermo Scientific; Waltham, MA, USA). Following DNA extraction, libraries were prepared by amplifying the 16s rRNA hypervariable regions V3 and V4 (341F CCTAYGGGRBGCASCAG and 806R GGACTACNNGGGTATCTAAT) with sequencing indexes, utilizing NEBNextR Ultra™ IIDNA Library Prep Kit (Cat No. E7645) (Illumina, Euroclone s.p.a., Italy). Amplicons were sequenced on a Novaseq 6000 platform, (Illumina; San Diego, CA, USA) in 2 × 250 paired-end mode, for an intended depth of sequencing of 200,000 reads per sample.

The FASTQ files were deposited in the NCBI Sequence Read Archive (PRJNA1246572 for dogs classified with other diseases, and PRJNA1247267 for dogs healthy or with osteoarthritis).

### 2.5. Bioinformatic

The FASTQ files were annotated with the Quantitative Insights into Microbial Ecology 2 (QIIME 2) [28] using DADA2 for noise reduction, performing dereplication to 100% similarity clustering, and chimeras were identified and removed. Overall, a total of 63,003,270 reads with an average count per sample of 360,018 reads (lowest count of 188,460 reads, highest 514,750 reads). De-duplicated sequences (Amplicon Sequence Variants, ASV) were reported to the feature table. Following DADA2, QIIME2’s classify-sklearn algorithm, a pre-trained Naive Bayes classifier, was applied for taxa annotation against the greengenes database 2022.10.backbone.full-length.nb.qza (https://ftp.microbio.me/greengenes_release/2022.10/) (accessed on 10 January 2025) [29] at the levels of kingdom, phyla, class, order, family, genus, and species were obtained.

ASVs were also annotated to the latest 2024.09 backbone.full-length.nb (https://ftp.microbio.me/greengenes_release/current/) (accessed on 10 January 2025) [30] classifier to take into account the recent new classification of the International Code of Nomenclature of Prokaryotes (ICNP, https://www.the-icsp.org/index.php/code-of-nomenclatur) (accessed on 10 January 2025) [31].

### 2.6. Statistical Analysis

The colony of Beagle dogs was grouped into Junior, Adult and Senior categories (age factor), considering the range of age: from 20 to 46 (Junior, 43 dogs), from 47 to 92 (Adult, 58 dogs) and more than 92 (Senior, 74 dogs). The first computation investigated the effect of age and included all the 175 dogs (Table 1). Considering that some of the dogs suffered from murmur, diabetes and other non-communicable disease, a second study involved only healthy or osteoarthritic (OA) animals. The number of dogs for Junior, Adult and Senior groups accounted for 30, 44 and 76, respectively (Table 2). All Junior dogs were healthy, while 23 Adult and 52 Senior dogs were affected by OA. Among the OA dogs, 1 Adult and 5 Seniors were also affected by other diseases and were not considered in this analysis. Arthrosis is a common disorder during aging and the Adult and Senior dogs were mildly affected and without severe pain, and no NSAIDs were administered during the 4 weeks before fecal sampling.

Taxonomic data were uploaded to the Microbiome Analyst (https://www.microbiomeanalyst.ca/, accessed on 31 January 2025) [32] for statistical analysis (Lu et al., 2023) [33] and normalized as (RA) for the taxonomic levels based on total sum. Shannon and Chao1 alpha diversity indexes were calculated and the comparisons for the whole dataset (175 dogs, factor Age) or between healthy and OA (136 dogs) were tested with the Mann–Whitney test, with FDR adjustment based on the Benjamini–Hochberg procedure. Bray–Curtis beta diversity differences for the factors age and OA were also computed, and the results were visualized using Principal Coordinate Analysis (PCoA) plots. Permutational multivariate analysis of variance (PERMANOVA) was used to assess differences in microbiota composition. Linear Discriminant Analysis (LDA) Effect Size (LEfSe) was applied [34] to compare RA between Age and healthy and OA dogs.

## 3. Results

### 3.1. Rarefaction Curve and Relative Abundance of Whole Population

The rarefaction curves show that species richness increases with sequencing depth across all age groups, and Firmicutes were the most abundant taxa, followed by Bacteroidetes (Supplementary Figure S1). The relative abundance (RA) at the phylum level, annotated using the 2022 greengenes version, indicated a prevalence of Firmicutes and Bacteroidetes, followed by Fusobacteria, Proteobacteria, Actinobacteria, Tenericutes, and Deferribacteres (Supplementary Figure S2A). Using the latest version of the greengenes classifier from 2024, the ranking of phyla was Bacillota_A, Bacteroidota, Fusobacteriota, Bacillota_I, Pseudomonadota, Bacillota_C, Actinomycetota, and Deferribacteriota (Supplementary Figure S2B). However, considering that most of the data still refers to the previous classification, for comparison purpose the annotations of 2022 greengene versions were used.

### 3.2. Alpha Diversity

**Figure 1:**
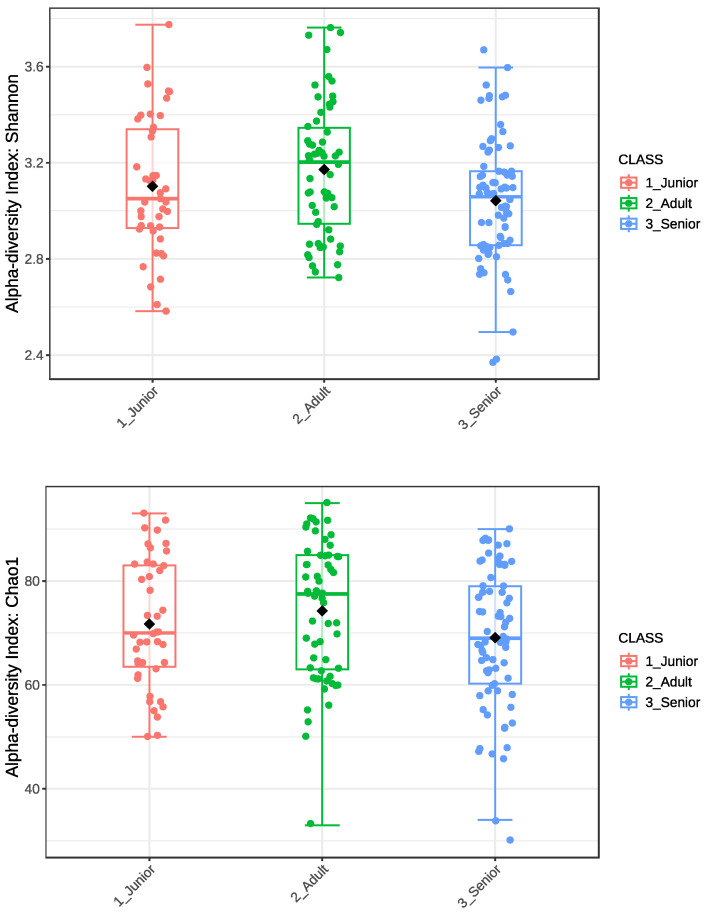
Alpha diversity index of Shannon and Chao1 of gut microbiota of the whole dog population (n = 175) grouped in Junior, Adult, and Senior based on age. Shannon index significantly differed between Adult and Senior for *p* < 0.05. Junior: dogs with age ranging 20–46 months; Adult: dogs with age ranging 47–92 months; Senior: dogs with age ranging 93–168 months.

Figure 1 presents the Shannon and Chao1 alpha diversity indices of the gut microbiota in the entire dog population (n = 175), categorized into Junior, Adult, and Senior groups. The boxplot illustrates the distribution of alpha diversity within each age group, highlighting median values, interquartile ranges, and individual data points. Significantly lower values (*p* < 0.05) were observed for the Shannon index in the Senior group compared to the Adult group.

**Figure 2:**
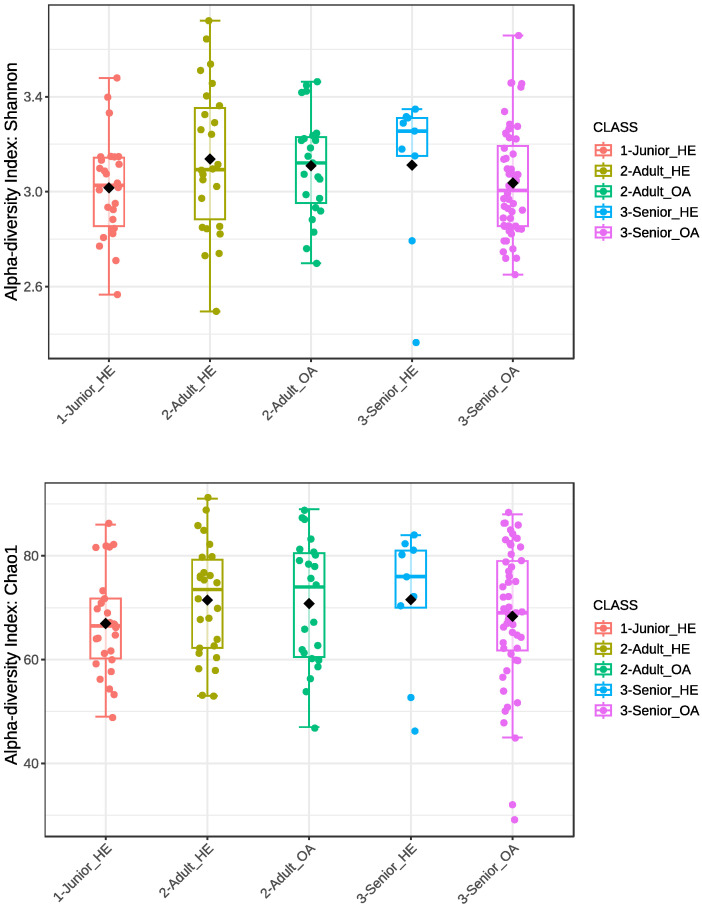
Alpha diversity index of Shannon and Chao1 of gut microbiota of selected healthy and osteoarthritic dogs grouped in Junior, Adult, and Senior based on age. Groups did not significantly differ for *p* < 0.05. Junior_HE: healthy dog with age ranging 20–46 months; Adult_HE: healthy dog with age ranging 47–92 months; Adult_OA: osteoarthritic dogs with age ranging 47–92 months; Senior_HE: healthy dogs with age ranging 93–168 months; Senior_OA: osteoarthritic dogs with age ranging 93–168 months.

The Shannon index and Chao1 indexes of alpha diversity were also analyzed to compare microbial diversity across different age groups and health conditions (Junior_HE, Adult_HE, Senior_HE, Adult_OA, and Senior_OA). As shown in Figure 2, variations in microbial diversity were observed among groups; however, no significant differences (*p* > 0.05) were detected for either the Shannon index or the Chao1 index. Despite this, the spread of values, indicated by the interquartile range and whiskers, showed high variability within each group. These results suggest that alpha diversity remains relatively stable across different age groups and health conditions in the studied dog population.

### 3.3. Beta Diversity

**Figure 3:**
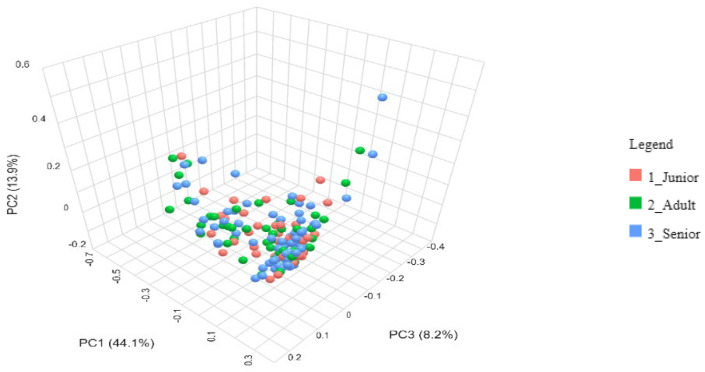
Bray-Curtis beta diversity index of gut microbiota of the dog population (number = 175) grouped in Junior, Adult, and Senior on the basis of age. Group of ages did not significantly differ for *p* < 0.05 at the PERMANOVA analysis. Junior: dogs with age ranging 20–46 months; Adult: dogs with age ranging 47–92 months; Senior: dogs with age ranging 93–168 months.

The beta diversity of the gut microbiota across different age groups of dogs was analyzed using the Bray–Curtis dissimilarity index and visualized through a Principal Coordinate Analysis (PCoA) plot (Figure 3). The distribution of data points representing Junior, Adult, and Senior dogs does not indicate distinct clustering among the age groups (*p* > 0.05). Instead, there is considerable overlap, suggesting that the gut microbiota composition remains relatively similar across different life stages.

**Figure 4:**
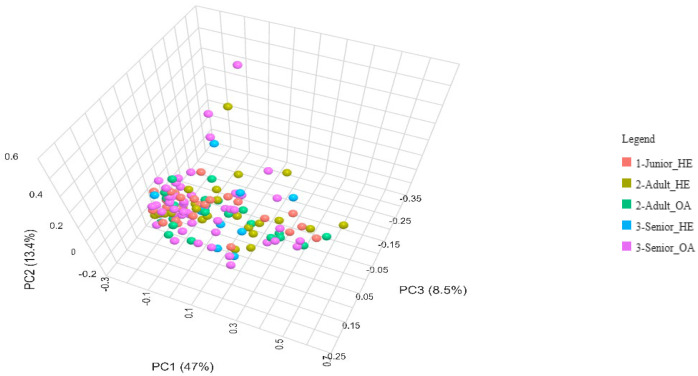
Bray-Curtis beta diversity index of gut microbiota of health and osteoarthritic dogs (n = 136) grouped in Junior, Adult, and Senior on the basis of age and healthy conditions. Groups did not significantly differ for *p* < 0.05 at the PERMANOVA analysis. Junior_HE: healthy dog with age ranging 20–46 months; Adult_HE: healthy dog with age ranging 47–92 months; Adult_OA: osteoarthritic dogs with age ranging 47–92 months; Senior_HE: healthy dogs with age ranging 93-168 months; Senior_OA: osteoarthritic dogs with age ranging 93–168 months.

The Bray–Curtis beta diversity index was also used to assess differences in gut microbiota composition among Junior_HE, Adult_HE, Senior_HE, Adult_OA, and Senior_OA dogs, and no significant differences (*p* > 0.05) were detected (Figure 4). The distribution of points did not form distinct clusters, indicating substantial overlap in the gut microbiota composition across all groups. Some outliers, represented by points positioned far from the main cluster, suggest that certain individuals have unique microbial compositions. The lack of significant differences implies that, despite aging and osteoarthritis, gut microbiota remains relatively stable in terms of beta diversity.

### 3.4. LEfSe Analysis

**Figure 5:**
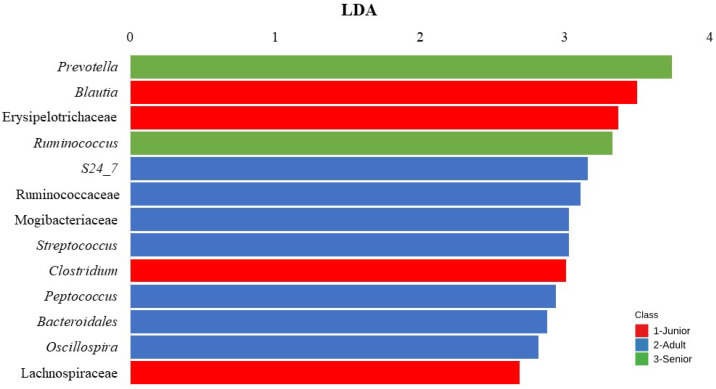
Result of Linear Discriminant Analysis (LDA) Effect Size of taxa which differ (FDR < 0.10; LDA score > 2) between age in the dog population (n = 175). Junior: dogs with age ranging 20–46 months; Adult: dogs with age ranging 47–92 months; Senior: dogs with age ranging 93–168 months.

Figure 5 presents a LEfSe analysis identifying bacterial taxa that were significantly different across the three age classes (Junior, Adult, and Senior) and in the Supplementary Table S1 the comparison of abundances between groups is reported. The LDA score (x-axis) quantifies the effect size of each significantly enriched bacterial taxon. The bacterial features (y-axis) represent taxa with an LDA score > 2 and an FDR < 0.10, indicating statistical significance. The results show that different bacterial taxa were associated with specific age groups. In the Adult group, the taxa *S24_7*, Ruminococcaceae, Mogibacteriaceae, *Streptococcus*, *Peptococcus*, Bacteroidales and *Oscillospira* significantly differed from the other groups.

**Figure 6:**
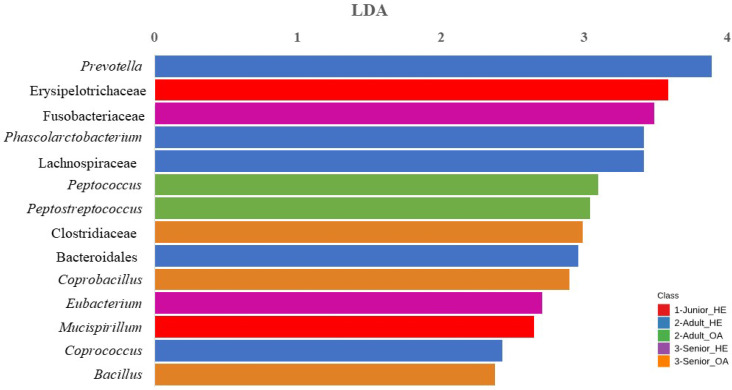
Result of Linear Discriminant Analysis (LDA) Effect Size of taxa which differs (FDR < 0.10; LDA score > 2) between age in healthy and osteoarthritic dog population (n = 136). Junior_HE: healthy dog with age ranging 20–46 months; Adult_HE: healthy dog with age ranging 47–92 months; Adult_OA: osteoarthritic dogs with age ranging 47–92 months; Senior_HE: healthy dogs with age ranging 93–168 months; Senior_OA: osteoarthritic dogs with age ranging 93–168 months.

In the Junior group, *Blautia*, Erysipelotrichaceae, *Clostridium* and Lachnospiraceae were significantly enriched. For the Senior group, *Prevotella* and *Ruminococcus* were identified as the most enriched taxa. The bar chart in Figure 6 represents the LDA of bacterial taxa that significantly differs (FDR < 0.10; LDA score > 2) across Junior_HE, Adult_HE, Senior_HE, Adult_OA, and Senior_OA dogs. In the Supplementary Table S2 the comparison of abundances between groups is reported. Junior_HE (healthy young dogs, red) was notably associated with Erysipelotrichaceae and *Mucispirillum*. Adult_HE (healthy Adult dogs, blue) had a higher RA of *Prevotella*, Bacteroidales, *Phascolarctobacterium*, Lachnospiraceae, Bacteroidales and *Coprococcus*. Adult_OA (osteoarthritic Adult dogs, green) showed significant associations with *Peptococcus* and *Peptostreptococcus*, indicating a shift in microbiota linked to osteoarthritis in Adult dogs. Senior_HE (healthy Senior dogs, purple) was dominated by Fusobacteriaceae and *Eubacterium*, and Senior_OA (osteoarthritic Senior dogs, orange) had a higher abundance of Clostridiaceae, *Coprobacillus*, and *Bacillus*.

## 4. Discussion

The composition of gut microbiota is essential for host health and is influenced by factors such as age and disease conditions. This study examined gut microbiota diversity in dogs across different life stages (Junior, Adult, and Senior) and health conditions (healthy vs. osteoarthritic).

### 4.1. Microbial Diversity

Shannon and Chao1 alpha diversity indices showed high individual variability across all age classes (Figure 1), with only the Adult dogs exhibiting a higher mean value for the Shannon index than the Senior dogs (*p* < 0.05). Mizukami et al. [35] reported a moderate decline in the alpha diversity of the gut microbiome with age, with both the Chao1 and Shannon indices showing a decreasing trend, although the changes were not statistically significant. However, they found that the Faith-PD index significantly decreased, indicating reduced phylogenetic diversity in aging dogs. Similarly, another study on the canine gut microbiome by Omatsu et al. [36] also reported a decrease in alpha diversity in aged dogs. In contrast, Fernández-Pinteño et al. [37] did not find notable differences in alpha diversity measures or the fecal dysbiosis index across age groups. The significant variation in the Shannon index and the stability of the Chao1 index observed in this study (Figure 1) could be attributed to the standardized dietary and environmental conditions of the dog population. Also, the comparison of healthy and OA dogs across the three age classes (Figure 2), after the exclusion of dogs with other pathologies, revealed a substantial stability of the bacterial community. Stevens et al. [38] did not find significant differences in the Shannon diversity index between healthy and OA pain dogs. Indeed, Cintio et al. [39] reported that the gut microbiota of OA dogs was significantly less diverse at the family taxonomic level compared to healthy dogs. The dogs in this latter study were client-owned and were fed the same diet for 45 days, conditions that differed from study of Stevens et al. [38], where privately owned dogs were fed different diets.

The lack of variation in beta diversity among Junior, Adult, and Senior dogs (Figure 3) suggested that gut microbiota composition remains relatively stable throughout life. However, Fernández-Pinteño et al. [37] and Mizukami et al. [35] found a significant difference in beta diversity with age when using the unweighted UniFrac metric, but not with the weighted UniFrac metric or Bray–Curtis distances. A limited number of studies reported the effect of OA on gut microbiomes in dogs. Stevens et al. [38] did not find variations in beta diversity between healthy and OA dogs, supporting the results obtained (Figure 4), and suggesting that the disease does not induce major shifts in gut microbial communities. Indeed, Cintio et al. [39] observed significant differences in beta diversity at the family level between healthy and OA dogs. Studies investigating the effect of OA on the gut microbiome of dogs are still limited and warrant further investigation.

### 4.2. Microbial Signatures Associated with Age and OA

LEfSe analysis identified distinct microbial taxa enriched across the age groups, indicating age-related changes in gut microbiota composition (Figure 5). In Junior dogs, a higher abundance of Lachnospiraceae and *Blautia* was reported compared to Adult and Senior dogs [40]. This agrees with the present results, suggesting a healthy gut microbiota [41,42].

An increase in Bacteroidales in Adult dogs could indicate immune homeostasis in the gut, as they are involved in carbohydrate degradation and the production of short-chain fatty acids [43]. The diverse family Erysipelotrichaceae, more abundant in Junior dogs, has been correlated with dietary carbohydrate and fiber digestion as well as short-chain fatty acid production [44]. This family has also been associated with healthier microbiota in dogs compared to those affected by enteropathies [42,45].

Fernández-Pinteño et al. [37] observed that S24-7 did not vary significantly with age but was found in higher abundance in Adult dogs. Senior dogs, on the other hand, showed higher levels of *Prevotella* and *Ruminococcus*, suggesting microbiota adaptations linked to the aging process. This is consistent with known microbial community shifts that occur as dogs age [37]. However, the variation in relative abundances (RAs) of other taxa did not align with the present study.

Mizukami et al. [35] found no significant correlation between aging and microbial changes at the genus or phylum levels. However, they identified a single ASV that showed a significant positive correlation with aging, suggesting the possibility of more subtle age-related shifts in the microbiome. This is in line with findings in humans, where certain microbes like *Absiella dolichum* have been found to be elevated in frail individuals [46] and lower in those on a high-fiber diet [47].

### 4.3. Microbiota and Osteoarthritis

When the data were analyzed separately by age class in healthy and OA dogs, higher relative abundances (RAs) of Erysipelotrichaceae and Bacteroidales were confirmed in Junior and Adult dogs, respectively (Figure 6). Interestingly, Fusobacteriaceae was more abundant in healthy Senior_HE dogs. You and Kim [48] also reported that the *Fusobacterium* genus was more abundant in older dogs (6–10 years) compared to younger dogs (0.5–1 year).

For healthy dogs (Junior_HE, Adult_HE, Senior_HE), the taxa that significantly changed did not correspond with those presented in Figure 5, suggesting that OA may influence the gut microbiome, potentially through inflammation-mediated changes. Notably, none of the Junior dogs were affected by OA, and the differences in taxa that appeared significantly higher in the analysis of the entire dataset may be attributed to statistical factors.

Research by Cintio et al. [39] found that OA dogs had an elevated relative abundance (RA) of the Megamonas genus and reduced levels of the Paraprevotellaceae, Porphyromonadaceae, and Mogibacteriaceae families compared to healthy dogs, indicating that dysbiosis or a disruption of the normal gut microbial balance could occur. In the study by Stevens et al. [38], dogs with OA showed higher representations of specific species, including *Bacteroides vulgatus*, *Eubacterium dolichum*, *Collinsella stercoris*, *Clostridium ramosum*, and *Ruminococcus torques*. The study did not report notable differences in the Firmicutes to Bacteroidetes ratio or in factors such as pain severity, mobility, activity level, age, or body composition score.

In the present study, the gut microbiota of Adult_OA dogs showed increased levels of *Peptococcus* and *Peptostreptococcus*, while that of Senior_OA dogs showed increased levels of Clostridiaceae, *Coprobacillus*, and *Bacillus* (Figure 6).

The role of specific microbial taxa in OA has also been investigated in human studies. Boer et al. [49] identified a significant association between *Streptococcus* species abundance in stool and OA-related knee pain and inflammation. Similarly, Yu et al. [50] suggested that specific microbial families and orders, such as Methanobacteriaceae and Desulfovibrionales, might play a causal role in OA development.

Despite the lack of strong evidence linking gut dysbiosis to OA in dogs, Stevens et al. [38] suggest that subtle changes in the gut microbiota could influence microbial product translocation and intestinal permeability. Elevated levels of lipopolysaccharide (LPS) and LPS-binding protein (LBP) are associated with increased OA severity in humans, and preliminary data from the same group suggests a positive correlation between LBP levels and the number of joints affected by OA-related pain in dogs. This indicates that intestinal permeability may play a critical role in OA progression and warrants further investigation [51].

Although some changes in fecal microbial population was observed, the limited variation in microbiota between class of ages and between healthy and OA dogs can be due to the relatively homogeneity of dog population and to the controlled environmental conditions, since dogs were fed the same thing and lived in similar quarters and with the same lifestyle. Limited variations in fecal microbiome in colony dogs were also reported by Mizukami et al. [35] and Uchiyama et al. [52]. The limitations of the study are the imbalance between healthy and OA groups especially in Seniors, along with the lack of measurements for markers of intestinal permeability (e.g., proinflammatory cytokines or LPS). The research provides support for new studies involving different breeds other than Beagle and other age-related diseases other than OA.

## 5. Conclusions

Our study confirmed that the gut microbiota undergoes distinct age-related changes in dogs, with different taxa becoming enriched or depleted across life stages, even though our results did not always align with those reported in the literature. While age influenced specific bacterial taxa, the overall microbial composition remained largely unchanged. OA appeared to induce microbial shifts in Adult and Senior dogs but did not significantly alter global diversity. These findings emphasize the need to explore the complex dynamics of microbial communities in aging dogs, particularly in relation to health conditions such as OA. Further studies with larger sample sizes and more diverse breeds are warranted to confirm these observations and gain a deeper understanding of the microbiome’s role in aging and disease.

## Figures and Tables

**Table 1:** Contingency table of the whole dog population by age, sex and healthy conditions.

Age	Sex	Healthy	Osteoarthritis	Other	Total
Junior	Fs	11	0	0	11
Adult		17	16	2	35
Senior		4	41	7	52
Junior	Mc	19	0	13	32
Adult		13	8	2	24
Senior		5	16	1	21
Junior	Total	30	0	13	43
Adult		30	24	4	58
Senior		9	57	8	74
Total		69	81	25	175

Fs: Spayed females; Mc: Castrated males; Junior: dog with age ranging 20–46 months; Adult dog with age ranging 47–92 months; Senior: dogs with age ranging 93–168 months; Other: 9 Junior dogs with diabetes and 4 Junior dogs with adverse drug reaction; 3 Adult dogs with seizure and 1 Adult dog with Cranial Cruciate Tear; 8 Senior dogs were affected by different diseases, as heart murmur (2), atopic dermatitis (1), congestive heart disease (1), seizure (4).

**Table 2:** Contingency table by age, sex and healthy conditions of the selected dog population.

		Healthy	Osteo-Arthritic		Total
	Fs	Mc	Fs	Mc	
Junior	10	16	0	0	26
Adult	14	12	16	7	49
Senior	4	5	37	15	61
Total	28	33	53	22	136

Junior: dogs with age ranging 20–46 months; Adult: dogs with age ranging 47–92 months; Senior: dogs with age ranging 93–168 months.

## Data Availability

The raw sequence data obtained was deposited in the NCBI Sequence. Read archive under the accession number PRJNA1246572 and PRJNA1247267.

## Supplementary Materials

The following supporting information can be downloaded at: https://www.mdpi.com/article/10.3390/ani15111619/s1, Figure S1: Rarefaction curves at the phylum level of gut microbiota of the 175 dogs Junior, Adult, and Senior based on age; Figure S2: Relative abundances at the phylum level of gut microbiota of the 175 dogs grouped in Junior, Adult, and Senior based on age; Figure S3: Result of Linear Discriminant Analysis (LDA) Effect Size of taxa which differ (FDR < 0.01; LDA score > 2) between age in the dog population (n = 175); Figure S4: Result of Linear Discriminant Analysis (LDA) Effect Size of taxa which differ (FDR < 0.01; LDA score > 2) between age in healthy and osteoarthritic dog population (n = 136).Table S1: Result of Linear Discriminant Analysis (LDA) Effect Size of taxa which differ (FDR < 0.10; LDA score > 2) between age in the dog population (n = 175); Table S2: Result of Linear Discriminant Analysis (LDA) Effect Size of taxa which differ (FDR < 0.10; LDA score > 2) between age in healthy and osteoarthritic dog population (n = 136).
