# Supplementary material for: Gut Microbiota Variation in Aging Dogs with Osteoarthritis
Source: Animals (Basel). 2025 May 30;15(11):1619. doi: 10.3390/ani15111619 (PMC12153520; doi:10.3390/ani15111619)
Supplement: Supplementary file 1 [file animals-15-01619-s001.zip › animals-3619845-supplementary.pdf]

Supplementary information

The Linear Discriminant Analysis (LDA) Effect Size (LEfSe) results in Supplementary Figure 4 highlight significant differences in gut microbial taxa across different age groups in a dog population (n=175). The taxa displayed have an LDA score >2 and FDR<0.01, indicating their relevance in distinguishing between age groups: Junior (20–46 months, red bars), Adult (47–92 months, blue bars), and Senior (93–168 months, green bars).

Supplementary Figure S3.

Microbiota and ageing

Junior dogs are enriched in *Blautia_A_141781, Thomasclavelia, Faecalibacillus, Anaerofilum_73741, Bacilli_A*, and others. These taxa are commonly associated with a younger gut microbiome, potentially aiding in digestion and metabolic activity. Adult dogs are characterized by higher levels of *Limosoma, Holdemanella, Negativibacillus, Eubacterium_B, Eisenbergiella, Onthovivens,* and *Anaerohabdus*. Many of these bacteria are involved in gut homeostasis and fermentation, suggesting a stable microbial environment in adulthood and Senior dogs are significantly enriched in *Clostridiaceae_222000* and *Mediterraneibacter_A_155507*. Some members of *Clostridiaceae* have been linked to shifts in gut microbial balance in aging individuals, possibly affecting nutrient absorption and immune function.

The LDA and LEfSe results presented in Figure 6 illustrate taxa that significantly differ between different age groups in healthy and osteoarthritic (OA) dogs. The taxa shown have an LDA score >2 and FDR<0.01, indicating significant differences in microbial composition across groups. The most discriminative taxa with the highest LDA scores vary across different groups, reflecting distinct gut microbiota compositions. In Healthy Adult (Adult_HE) dogs, key enriched taxa include *Prevotella, Lachnospiraceae*, and *Phascolarctobacterium_A_39470*, which are associated with beneficial fermentation processes and overall gut health. In contrast, Osteoarthritic Adult (Adult_OA) dogs show increased levels of *Peptostreptococcus* and *Faecalibaculum*, both of which have been linked to inflammatory processes and altered gut metabolism.

In younger dogs, the Junior Healthy (Junior_HE) group is primarily characterized by *Faecalibacillus*, which may indicate a microbiota composition typical of younger individuals. Meanwhile, Healthy Senior (Senior_HE) dogs exhibit an enrichment of *Escherichia, Negativibacillus*, and *Lachnospira*, suggesting age-related shifts in the gut microbiome.

The progression of gut microbiota across different life stages reflects microbial maturation and aging trends, shaping metabolic and immune functions over time. Younger dogs (Junior group) show an enrichment of taxa linked to rapid metabolism and energy processing, likely due to their higher activity levels and dietary requirements. Adult dogs exhibit a more balanced microbial composition, with bacteria supporting gut stability, fermentation, and immune

regulation. Senior dogs display a shift towards taxa that may influence gut dysbiosis and metabolic changes, which could be associated with age-related immune or digestive alterations. The increased presence of *Clostridiaceae* and *Mediterraneibacter* in senior dogs could indicate gut microbiome alterations related to aging, such as reduced microbial diversity or changes in fiber digestion. The presence of *Negativibacillus* and *Holdemanella* in adult dogs suggests a mature and functionally diverse microbiota, contributing to metabolic homeostasis. The abundance of *Blautia* and *Faecalibacillus* in juniors highlights the presence of species that may contribute to nutrient absorption and immune development in younger dogs.

Supplementary Figure S4.

Microbiota and Osteoarthritis

The LDA analysis reveals striking differences in gut microbiota composition across different age groups and osteoarthritic conditions, highlighting the intricate relationship between microbiota, aging, and disease progression in dogs. Healthy adult dogs exhibit a diverse and well-balanced microbiome, enriched with beneficial taxa such as *Prevotella, Lachnospiraceae,* and *Phascolarctobacterium_A_39470*. This microbial richness supports gut stability, fermentation, and immune regulation, contributing to overall health. However, as osteoarthritis develops, a distinct microbial shift occurs. Osteoarthritic adult dogs experience microbial dysbiosis**,** with an increased presence of *Peptostreptococcus*, a taxon often associated with inflammation and disease progression**.** This disruption in microbial balance may exacerbate gut-derived inflammation and metabolic disturbances**,** potentially playing a role in osteoarthritis pathology.

As dogs age, their microbiota composition continues to evolve, reflecting age-related physiological changes. Senior dogs exhibit a marked shift towards bacteria associated with metabolic alterations and immune function decline**.** The enrichment of *Escherichia* and *Negativibacillus* in senior healthy dogs suggests potential impairments in gut homeostasis, while the increased presence of *Clostridiaceae* and *Mediterraneibacter* may indicate reduced microbial diversity and altered fiber digestion**.** These microbial changes could influence nutrient absorption, immune regulation, and gut barrier integrity, making senior dogs more susceptible to metabolic imbalances and inflammatory conditions.

Supplementary Figure S1. Rarefaction curves at the phylum level of gut microbiota of the 175 dogs Junior, Adult and Senior based on age.

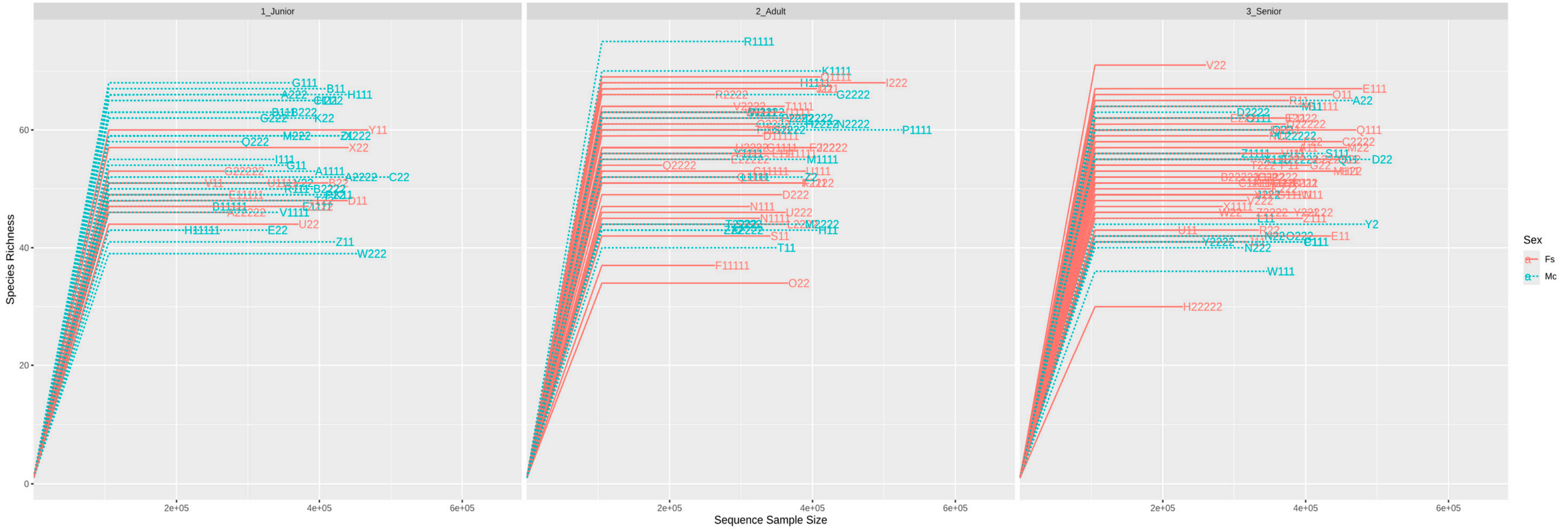

1-Junior: dog with age ranging 20–46 months (43 dogs); 2-Adult dog with age ranging 47–92 months (58 dogs); 3-Senior: dogs with age ranging 93–168 months (74 dogs).

Supplementary Figure S2. Relative abundances at the phylum level of gut microbiota of the 175 dogs grouped in Junior, Adult and Senior based on age.

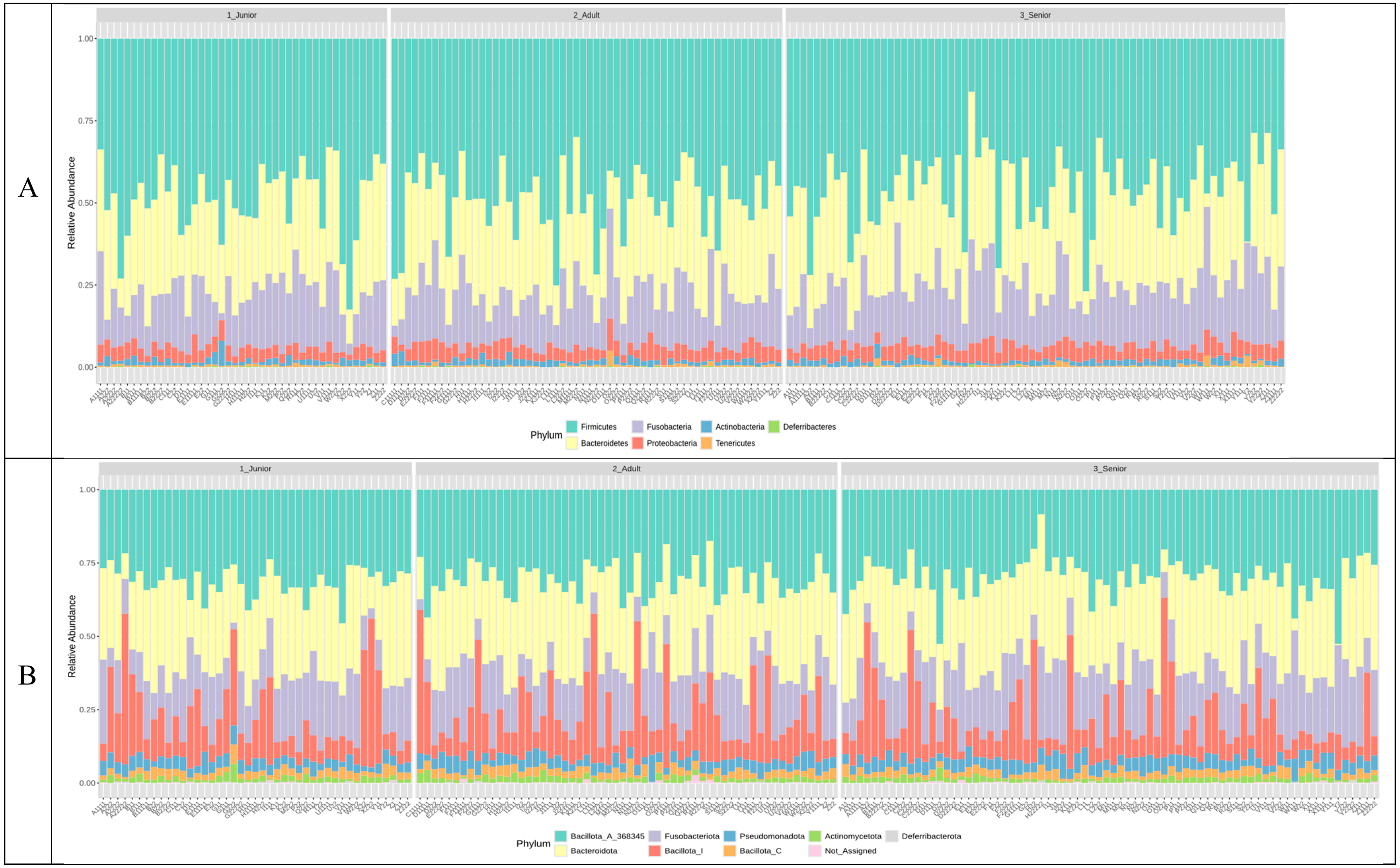

Bacillota A, Bacteroidota; Fusobacteriota, Bacillota_I Pesudomonadota, Bacillota_C; Actinomycetota and Deferribacteriota;1-Junior: dog with age ranging 20–46 months (43 dogs); 2-Adult dog with age ranging 47–92 months (58 dogs); 3-Senior: dogs with age ranging 93–168 months (74 dogs).

Supplementary Figure S3. Result of Linear discriminant analysis (LDA) effect Size of taxa which differ (FDR<0.01; LDA score >2) between age in the dog population (n=175).

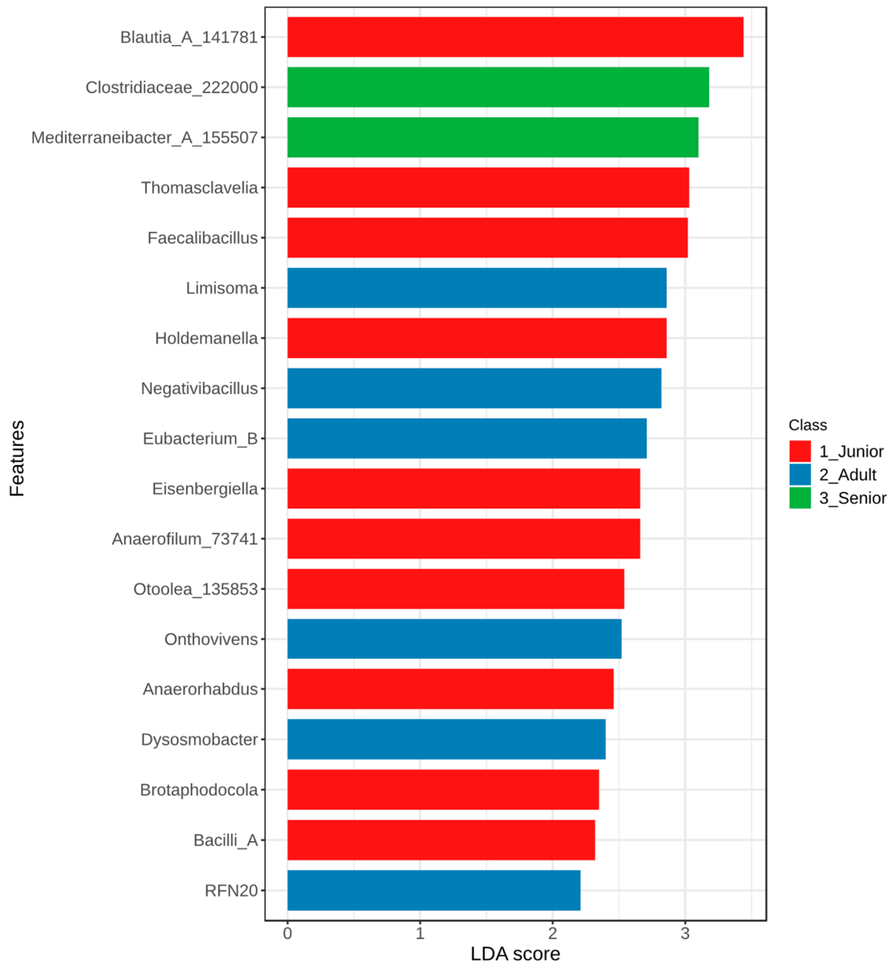

Junior: dog with age ranging 20–46 months; Adult dog with age ranging 47–92 months; Senior: dogs with age ranging 93–168 months.

Supplementary Figure S4. Result of Linear discriminant analysis (LDA) effect Size of taxa which differ (FDR<0.01; LDA score >2) between age in healthy and osteoarthritic dog population (n=136)

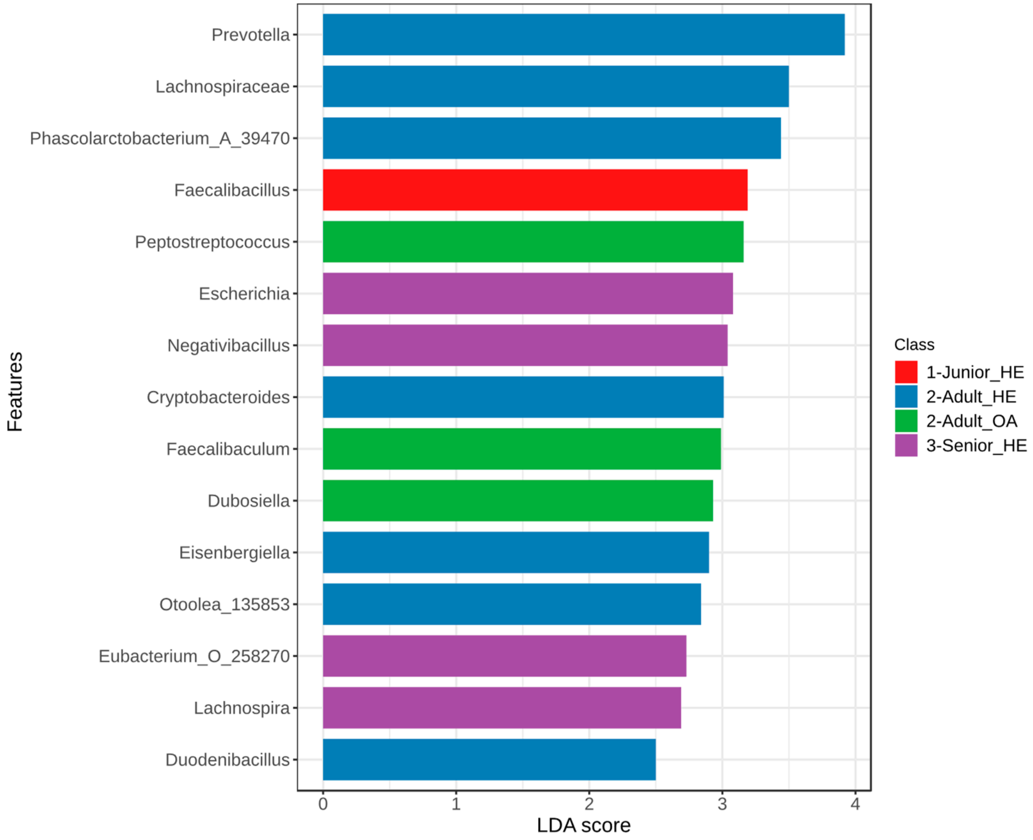

Junior_HE: healthy dog with age ranging 20–46 months; Adult_HE: healthy dog with age ranging 47–92 months; Adult_OA: osteoarthritic dogs with age ranging 47–92 months; Senior_HE: healthy dogs with age ranging 93–168 months; Senior_OA: osteoarthritic dogs with age ranging 93–168 months.

Supplementary Table S1. Result of Linear discriminant analysis (LDA) effect Size of taxa which differ (FDR <0.10; LDA score >2) between age in the dog population (n=175).

Taxa	FDR	LDAscore	Junior	Adult	Senior
Prevotella	0.083	3.7	29342	33464	22414
Blautia	0.018	3.5	36717	33561	30427
Erysipelotrichaceae	0.001	3.4	13603	10050	8916
Ruminococcus	0.018	3.3	11858	15342	16092
S24_7	0.084	3.2	10058	11232	8361
Ruminococcaceae	0.006	3.1	8083	9457	6888
Mogibacteriaceae	0.109	3.0	4460	5575	3449
Streptococcus	0.070	3.0	1061	3212	2999
Clostridium	0.332	3.0	4573	3368	2517
Peptococcus	0.094	2.9	2835	4560	2884
Bacteroidales	0.071	2.9	1460	2875	1359
Oscillospira	0.093	2.8	3227	4183	2849
Lachnospiraceae	0.049	2.7	5922	5560	4938

Junior: dog with age ranging 20–46 months; Adult dog with age ranging 47–92 months; Senior: dogs with age ranging 93–168 months.

Supplementary Table S2. Result of Linear discriminant analysis (LDA) effect Size of taxa which differ (FDR <0.10; LDA score >2) between age between age in healthy and osteoarthritic dog population (n=136).

Taxa	FDR	LDA	1-Junior_HE	2-Adult_HE	2-Adult_OA	3-Senior_HE	3-Senior_OA
Prevotella	0.049	3.9	20992	35809	30127	30508	20435
Erysipelotrichaceae	0.049	3.6	13495	11127	9457	5671	10346
Fusobacteriaceae	0.067	3.5	6328	5523	9601	11641	6574
Phascolarctobacterium	0.009	3.4	18050	21929	21307	17449	16636
Lachnospiraceae	0.075	3.4	15600	19364	16204	14121	16086
Peptococcus	0.065	3.1	1611	3893	4115	3631	2830
Peptostreptococcus	0.021	3.0	655	968	2870	974	1347
Clostridiaceae	0.062	3.0	2985	1738	1247	3168	3190
Bacteroidales	0.078	3.0	437	2251	2101	1271	1428
Coprobacillus	0.045	2.9	2012	2969	1503	2449	3092
Eubacterium	0.063	2.7	484	844	1170	1513	606
Mucispirillum	0.035	2.7	1091	190	953	562	1061
Coprococcus	0.095	2.4	415	957	724	539	812
Bacillus	0.022	2.4	615	496	428	258	741

Junior_HE: healthy dog with age ranging 20–46 months; Adult_HE: healthy dog with age ranging 47–92 months; Adult_OA: osteoarthritic dogs with age ranging 47–92 months, Senior_HE: healthy dogs with age ranging 93–168 months; Senior_OA: osteoarthritic dogs with age ranging 93–168 months.
